# Urinary cGMP predicts major adverse renal events in patients with mild renal impairment and/or diabetes mellitus before exposure to contrast medium

**DOI:** 10.1371/journal.pone.0195828

**Published:** 2018-04-12

**Authors:** Lyubov Chaykovska, Fabian Heunisch, Gina von Einem, Carl-Friedrich Hocher, Oleg Tsuprykov, Mira Pavkovic, Peter Sandner, Axel Kretschmer, Chang Chu, Saban Elitok, Johannes-Peter Stasch, Berthold Hocher

**Affiliations:** 1 Center for Cardiovascular Research, Charité Universitaetsmedizin Berlin, Berlin, Germany; 2 Department of Vascular Surgery, University Hospital Zurich, Zurich, Switzerland; 3 Institute for Nutritional Science, University of Potsdam, Potsdam, Germany; 4 Bayer AG, Biomarker Research, Wuppertal, Germany; 5 Departments of Nephrology, The First Affiliated Hospital, Jinan University, Guangzhou, China; 6 Department of Nephrology and Endocrinology/Diabetology, Klinikum Ernst von Bergmann, Potsdam, Germany; 7 Department of Pharmacology, School of Pharmacy, Martin-Luther-University, Halle-Wittenberg, Germany; 8 Bayer AG, Cardiovascular Research, Wuppertal, Germany; 9 Key Laboratory of Study and Discovery of Small Targeted Molecules of Hunan Province, School of Medicine, Hunan Normal University, Changsha, China; 10 Key Laboratory for Regenerative Medicine of the Ministry of Education, Division of Histology and Embryology, Medical College, Jinan University, Guangzhou, China; The University of Tokyo, JAPAN

## Abstract

**Background:**

The use of iodine-based contrast agents entails the risk of contrast induced nephropathy (CIN). Radiocontrast agents elicit the third most common cause of nephropathy among hospitalized patients, accounting for 11–12% of cases. CIN is connected with clinically significant consequences, including increased morbidity, prolonged hospitalization, increased risk of complications, potential need for dialysis, and increased mortality rate. The number of in-hospital examinations using iodine-based contrast media has been significantly increasing over the last decade. In order to protect patients from possible complications of such examinations, new biomarkers are needed that are able to predict a risk of contrast-induced nephropathy. Urinary and plasma cyclic guanosine monophosphate (cGMP) concentrations are influenced by renal function. Urinary cGMP is primarily of renal cellular origin. Therefore, we assessed if urinary cGMP concentration may predict major adverse renal events (MARE) after contrast media exposure during coronary angiography.

**Methods:**

Urine samples were prospectively collected from non-randomized consecutive patients with either diabetes or preexisting impaired kidney function receiving intra-arterial contrast medium (CM) for emergent or elective coronary angiography at the Charité Campus Mitte, University Hospital Berlin. Urinary cGMP concentration in spot urine was analyzed 24 hours after CM exposure. Patients were followed up over 90 days for occurrence of death, initiation of dialysis, doubling of plasma creatinine concentration or MARE.

**Results:**

In total, 289 consecutive patients were included into the study. Urine cGMP/creatinine ratio 24 hours before CM exposure expressed as mean±SD was predictive for the need of dialysis (no dialysis: 89.77±92.85 μM/mM, n = 277; need for dialysis: 140.3±82.90 μM/mM, n = 12, p = 0.008), death (no death during follow-up: 90.60±92.50 μM/mM, n = 280; death during follow-up: 169.88±81.52 μM/mM, n = 9; p = 0.002), and the composite endpoint MARE (no MARE: 86.02±93.17 μM/mM, n = 271; MARE: 146.64±74.68 μM/mM, n = 18, p<0.001) during the follow-up of 90 days after contrast media application. cGMP/creatinine ratio stayed significantly increased at values exceeding 120 μM/mM in patients who developed MARE, required dialysis or died.

**Conclusions:**

Urinary cGMP/creatinine ratio ≥ 120 μM/mM before CM exposure is a promising biomarker for the need of dialysis and all-cause mortality 90 days after CM exposure in patients with preexisting renal impairment or diabetes.

## Introduction

Iodinated radiographic contrast media can cause kidney dysfunction, particularly in patients with preexisting renal impairment and/or diabetes. This dysfunction may lead to additional kidney injury, a so-called contrast induced acute kidney injury (AKI) defined as an acute impairment of renal function and manifested by an absolute increase of serum creatinine of at least 0.5 mg/dL or by a relative increase by at least 25% from baseline levels [1]. Peak creatinine typically occurs 3–5 days after contrast media administration and returned to baseline (or a new baseline) within 1–3 weeks [2]. Contrast-induced AKI is associated with an increase in both short- and long-term morbidity and mortality, increased hospital length of stay, and greater health care costs [3].

Diabetes and chronic renal dysfunction are independent factors for the development of coronary artery disease. Percutaneous coronary interventions (PCI) became a routine procedure, and they are performed more and more frequently in patients with significant co-morbidities such as chronic kidney disease (CKD) and/or diabetes. In addition, patients with CKD and/or diabetes have an increased mortality after PCI with or without stenting [4]. Therefore, there is a high need to identify biomarkers associated with subsequent life-threatening complications after CM application.

Mechanisms underlying contrast media nephrotoxicity are multifactorial, including renal ischemia, particularly in the renal medulla, the formation of reactive oxygen species (ROS), reduction of nitric oxide (NO) production, and tubular epithelial and vascular endothelial injury [5]. NO and atrial natriuretic peptide (ANP) induce vascular relaxation by increasing the production of cGMP, an important mediator of vascular tone. Although NO generation in the kidney is essential for preservation of renal perfusion and function, high levels of NO secondary to an increase in iNOS activity may inhibit eNOS activity, resulting in renal vasoconstriction and decreased GFR [6]. Fractional urine excretion of ANP [7] is increased in patients with chronic renal failure and is significantly correlated with creatinine clearance [8–13]. cGMP is produced by particulate (pGC) and soluble (sGC) guanylyl cyclases, as a result of natriuretic peptide and NO activation [14]. Although both sGC and pGC activation increase cGMP intracellular concentration, pGC activation (unlike sGC) results also in significant release of cGMP into the extracellular space and blood circulation [15–19]. Therefore, while both sGC and pGC increase cGMP within cells, the resulting biological actions are quite different. Due to in part different localizations of sGC and pGC receptors, and timing of their signaling in the kidney, the NP/pGC/cGMP pathway predominantly regulates GFR and sodium excretion whereas the NO/sGC/cGMP pathway mostly controls renal vascular tone[20, 21]. Urinary and plasma cGMP concentrations are influenced by renal function [19, 22]. Urinary cGMP is primarily of renal cellular origin [17]. A positive correlation between increased cGMP in plasma and cGMP in urine was shown previously [23], therefore it was suggested as biomarker candidate for kidney injury. Furthermore, in contrast to plasma cGMP, urinary cGMP is not related to the severity of heart failure as assessed clinically according to the NYHA classification[19]. Thus, the goal of our study was to evaluate the predictive value of urinary cGMP clearance 24 hrs prior to contrast medium application for the development of adverse events in patients at risk for contrast-induced kidney injury.

## Methods

### 2.1 Study design

A prospective cohort of 289 consecutive patients underwent coronary angiography between January 2010 and December 2011 in the Department of Cardiology of the [13]Charité –Universitätsmedizin Berlin [24, 25]. This study was approved by the institutional review board and by the Ethics Commission of the Charité –Universitätsmedizin Berlin. Prior to enrollment into the study, each participant of the study has signed an informed consent form, which was approved by the Ethics Commission of the Charité –Universitätsmedizin Berlin. The study was conducted according to the Declaration of Helsinki, the European Guidelines on Good Clinical Practice and relevant national and regional authority requirements.

#### 2.1.1 Inclusion criteria

Consecutive patients with plasma creatinine of at least 1.1 mg/dL or preexisting diabetes mellitus were considered as potential study participants.

Consecutive patients with a high risk of developing contrast induced renal failure, i.e. patients with plasma creatinine levels of at least 1.1 mg/dL but not requiring dialysis or patients with preexisting diabetes mellitus independently of plasma creatinine levels, were enrolled into the study. Inclusion criteria were based on Mehran contrast nephropathy risk score[26].

#### 2.1.2 Exclusion criteria

Patients with end-stage renal disease as well as patients who were not able or refused to sign an informed consent were not included.

### 2.2 Course of the study (Fig 1)

All study participants underwent blood and urine sampling 24 hrs prior to coronary angiography with water-soluble, non-ionic, monomeric, low-osmolar, iodine-based contrast agent Iobitridol at a concentration of 350 mg Iod/mL (XENETIX® 350, Guerbet GmbH, Sulzbach/Taunus, Germany). Further, blood and urine samples were obtained 24 and 48 hrs after contrast agent application and 90 days after angiography [24].

**Fig 1:**
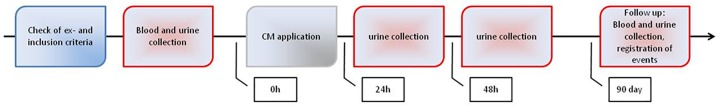
Flow chart for sample collection.

### 2.3 Sample handling and biomarker measurement

Blood samples were centrifuged for 5 min at 3.000 rpm and the resulting plasma was immediately frozen at -80°C. Creatinine was measured according to the Jaffé method. Glomerular filtration rate (GFR) was estimated according to the modification of diet in renal disease (MDRD) formula (eGFR = 175 x (SCr)-1.154 x (age)-0.203 x 0.742 [if female])[27]. cGMP concentrations were measured using a commercially available radioimmunoassay kit (IBL, Hamburg, Germany) as previously described [28].

### 2.4 Study endpoints (Table 1)

Study endpoints included death, initiation of dialysis, doubling of plasma creatinine concentration during the 90 days follow-up or major adverse renal events (MARE). MARE was defined as an occurrence of death, initiation of dialysis or doubling of the creatinine concentration within 90 days after the procedure. A predictive value for the risk to develop one of the endpoints was assessed based on urinary cGMP/creatinine ratios 24 hrs prior to contrast medium application. Additionally, urinary cGMP/creatinine ratios were calculated for 24 and 48 hrs after CM exposure. According to National Kidney Foundation–Kidney Disease Outcomes Quality Initiative (NKF-KDOQI) guideline, the definition of chronic kidney disease is a GFR below 60 ml/min/1,73 m2 for three months or more or a GFR above 60 ml/min/1,73 m2 with kidney damage marked by high levels of albumin in urine[29].

**Table 1:** Overview of study endpoints.

Endpoints	N	%
Death	9	3.1
Dialysis	12	4.1
Doubling of serum creatinine	1	0.3
MARE	18	6.2

N—number of patients, %—percentage of the patients in total study cohort, MARE—major adverse renal event

### 2.5 Statistical analysis

The statistical analysis was performed using SPSS 20 (IBM® SPSS® Statistics IBM Cooperation, Armonk, USA). Shapiro–Wilk test was used evaluate the distribution of the data. The predictive value of urinary cGMP/creatinine ratios as a biomarker of death or MARE was assessed by receiver operating characteristic (ROC) analysis.

Mean levels of urinary cGMP/creatinine ratios at different time points were compared using *t*-tests. Cumulative MARE rates were compared using a χ^2^ tests with risk ratios (with 95% confidence intervals). For all analyzes a two-sided t-test was used. P of less than 0.05 was considered statistically significant.

## Results

### 3.1 Patients characteristics

**Fig 2:**
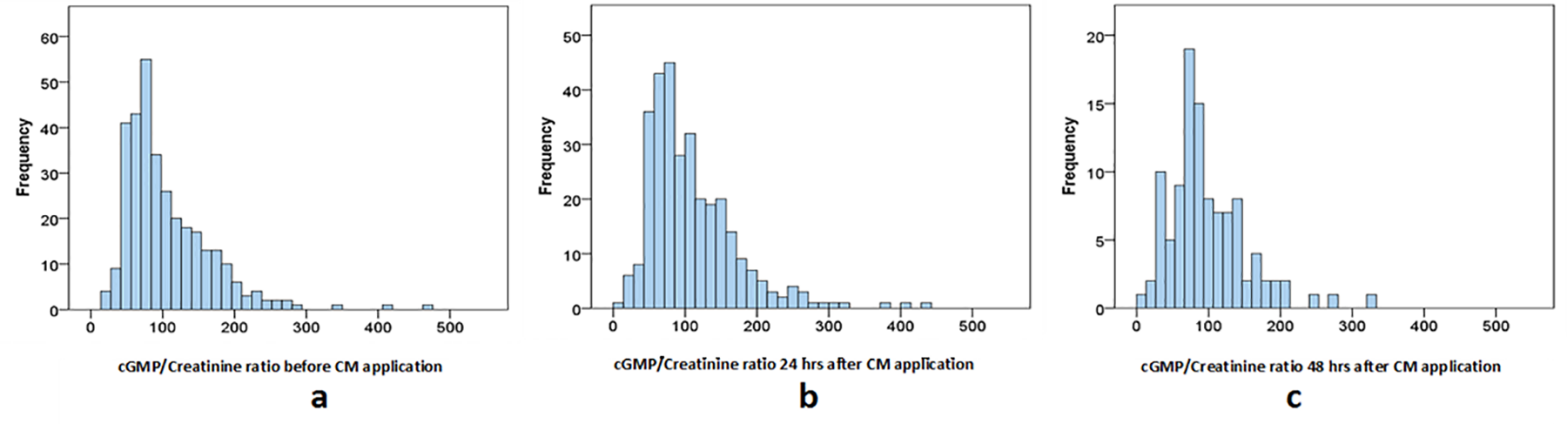
**Histogram of cGMP/Creatinine ratio (μM/mM) (a) before, (b) 24h after and (c) 48h after CM application.** *cGMP*: *urinary cyclic guanosine monophosphate*.

The study cohort included 289 consecutive patients who underwent coronary angiography (254 (77.0%) male and 76 (23.0%) female) with mean age of 68.91 ± 9.75 years and a body mass index (BMI) of 28.99 ± 5.57 kg/m^2^. Baseline creatinine was 1.24 ± 0.46 mg/dL, which corresponded to baseline GFR of 64.77 ± 21.73 ml/min/1.73m^2^. 156 patients (54.0%) patients were previously diagnosed with diabetes mellitus, 76 (26.3%) suffered from congestive heart failure and 74 (25.6%) had anemia. Anemia was diagnosed according to the definition of the World Health Organization: baseline hematocrit value <39% for men and <36% for women[30]. Most of the patients were treated with angiotensin-converting-enzyme (ACE) inhibitors or angiotensin II receptor blockers (ARBs), in combination with diuretics and/or statins. ACE inhibitors, ARBs and diuretics are known for causing an acute kidney injury (AKI) in CKD patients, therefor they were stopped in patients that developed AKI. None of the patients from our cohort used of nitroglycerin or natriuretic peptides in order to avoid possible effect of these agents on urinary cGMP excretion. Mean volume of injected contrast medium was 113.35 ± 55.02 mL (Table 2). Urinary cGMP/creatinine ratios in our patient cohort were normally distributed at each follow-up time point (Fig 2).

**Table 2:** Baseline characteristics of the cohort.

Patients characteristics	
Female N (%)/Male N (%)	76 (23.0)/254 (77.0)
Age, years (Median ± SD)	68.91 ± 9.75
Body mass index, kg/m^2^ (Median ± SD)	28.99 ±5.57
CM-volume, ml (Median ± SD)	113.35 ± 55.02
Baseline creatinine, mg/dl (Median ± SD)	1.24 ± 0.46
Baseline GFR, ml/min/1.73m^2^ (Median ± SD)	64.77 ± 21.73
Diabetes mellitus N (%)	156 (54.0)
Congestive heart failure N (%)	76 (26.3)
Anemia N (%)	74 (25.6)
Hypertension N (%)	257 (88.9)
Intake of N (%):	
*- ACE inhibitors*	161 (55.7)
*- ARB*	98 (33.9)
*- Diuretics*	185 (64.0)
*- Statins*	182 (63.0)

N—number of patients, %—percentage of the patients in the study cohort, CM—contrast media, GFR—glomerular filtration rate according to the MDRD formula, SD—standard deviation; ACE—angiotensin converting enzyme, ARB—angiotensin II receptor blocker

### 3.2 Correlation between cGMP and the study endpoints

**Fig 3:**
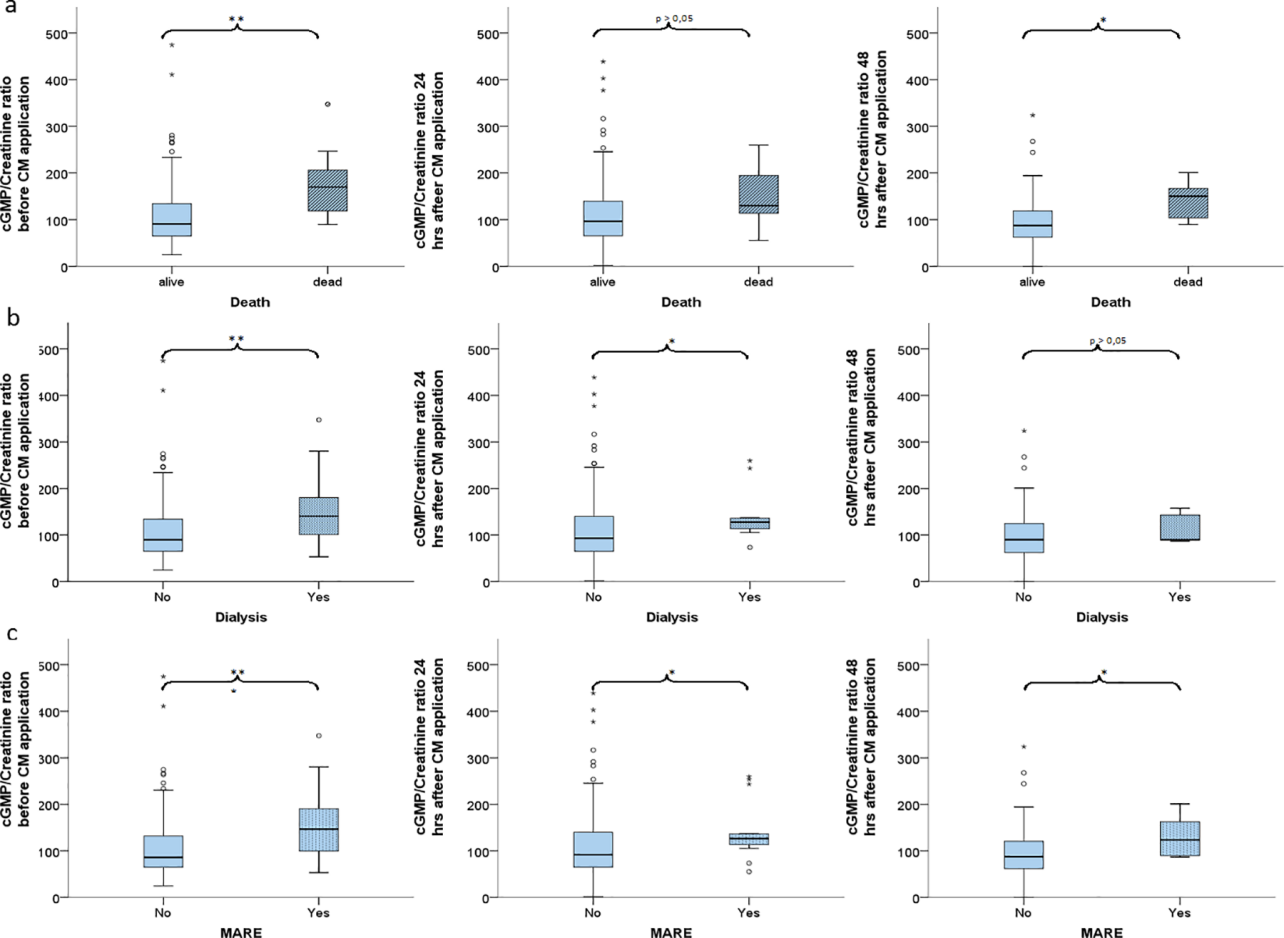
**Distribution of urinary cGMP/creatinine ratios (μM/mM) before 24 hours after and 48h after contrast media application detected in patients without (No) or with (Yes) following adverse events: death(a), dialysis (b) or MARE (c).** *cGMP*: *urinary cyclic guanosine monophosphate*, *MARE*: *major adverse renal event*, *** p<0.*001*, ** p<0.*01*, * p<0.*05*.

**Fig 4:**
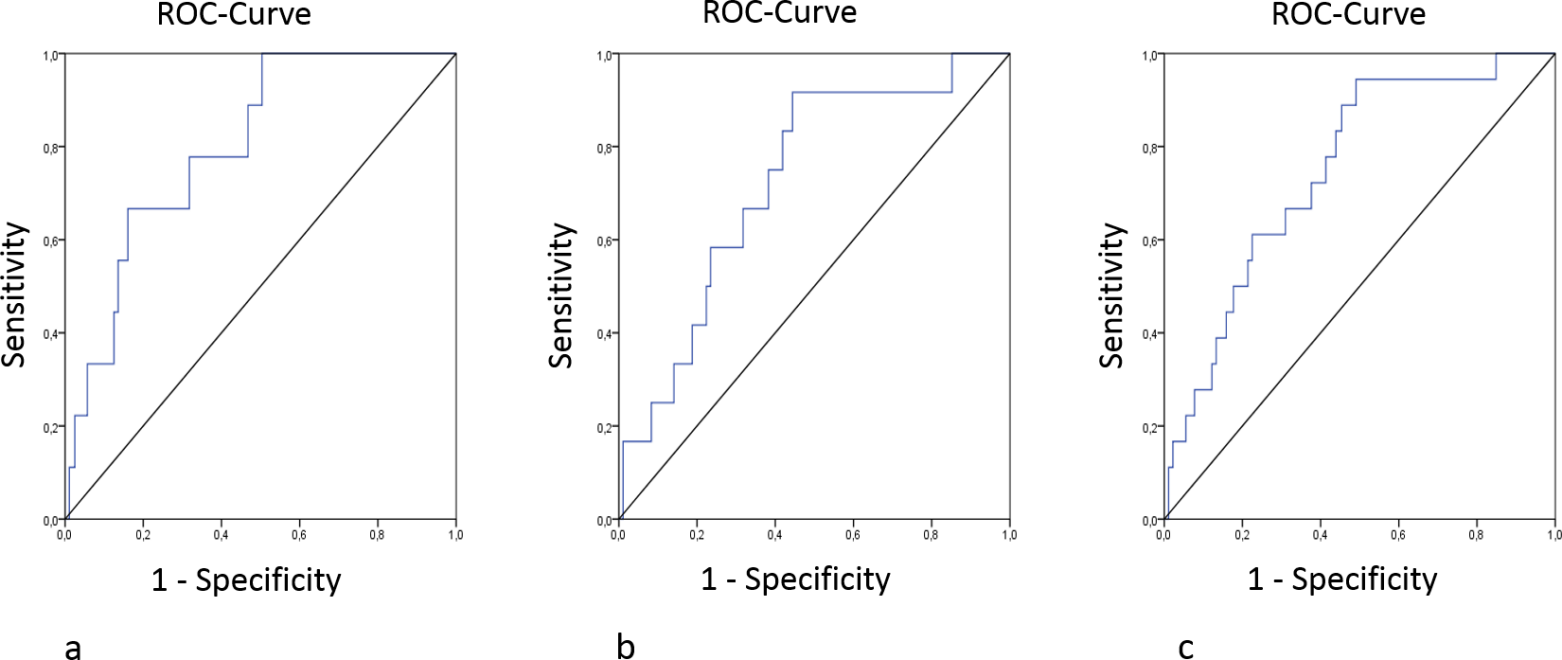
ROC-curves of the cGMP/Creatinine ratio before CM application for (a) death, (b) dialysis and (c) MARE.

Nine patients died during the follow-up time of 90 days. The average death occurred at 74.5 days (95% CI 7–95) after study entry. Causes of death included cardiovascular diseases in 4 patients, infections in 2 patients, respiratory failure in 1 patient and unknown reasons in 2 patients. Urinary cGMP/creatinine ratios were significantly lower before CM injection in survivors (90.60±92.50 μM/mM) compared with deceased patients (169.88±81.52 μM/mM, n = 9; p = 0.002). This difference remained significant at 48 hours after CM application (87.44±54.23 vs.150.23±41.26 μM/mM, p = 0.014) (Table 3, Fig 3A). ROC analysis confirmed that increased urinary cGMP/creatinine ratiois a significant predictor of death during 90 days after CM application in patients with plasma creatinine of at least 1.1 mg/dl or preexisting diabetes mellitus (Fig 4A).

**Table 3:** Ratios of urinary cGMP and creatinine (in μM/mM) before, 24 hours after and 48h after contrast media application.

		cGMP/Cr before CM			cGMP/Cr 24 hours after CM			cGMP/Cr 48 hours after CM		
		N	M ± SD	p	N	M ± SD	p	N	M ± SD	p
**MARE**	No	271	86.02 ± 93.17	<0.001	250	92.01 ± 62.29	0.014	85	87.44 ± 54.86	0.039
	Yes	18	146.64 ± 74.68		16	126.45 ± 59.67		8	123.37 ± 43.38	
**Dialysis**	No	277	89.77 ± 92.85	0.008	255	93.71 ± 62.48	0.028	88	89.88 ± 55.55	0.323
	Yes	12	140.30 ± 82.90		11	127.50 ± 57.14		5	89.74 ± 34.22	
**Death**	No	280	90.60 ± 92.50	0.002	258	96.81 ± 62.00	0.069	87	87.44 ± 54.23	0.014
	Yes	9	169.88 ± 81.52		8	129.91 ± 71.19		6	150.23 ± 41.26	

cGMP—urinary cyclic guanosine monophosphate, Cr—urinary creatinine, MARE—major adverse renal event; N—number of patients, M—median, SD—standard deviation, p—significance according to MANOVA, p<0.05 is statistically significant.

12 patients in our cohort needed dialysis during the follow-up period. cGMP/creatinine ratios in urine 24 hrs before CM injection were significantly higher in those patients (140.3±82.90 vs. 89.77±92.85 μM/mM, p = 0.008). This difference remained significant 24 hrs after CM application (127.50±57.14 vs. 93.71±62.48 μM/mM, p = 0.028) (Table 3, Fig 3B). ROC analysis showed that increased urinary cGMP/creatinine ratios are predictive for dialysis following CM application in patients with creatinine concentration of at least 1.1 mg/dL or preexisting diabetes mellitus (Fig 4B).

MAREs were detected in 18 patients of our study population and were characterized by significantly higher levels of urinary cGMP/creatinine ratio 24 hrs prior to CM exposure (146.64±74.68 vs. 86.02±93.17 μM/mM, p<0.001). This difference remained significant at 24 hrs (126.45±59.67 vs. 92.01±62.29 μM/mM, p = 0.014) and 48 hrs after coronary angiography (123.37±43.38 vs. 87.44±54.86 μM/mM, p = 0.039) (Table 3, Fig 3C). ROC curve demonstrated that an increase in urine cGMP/creatinine ratio strongly predicted the risk of MARE following CM application in patients with creatinine of at least 1.1 mg/dL or preexisting diabetes mellitus (Fig 4C). Multivariate logistic regression analysis confirmed that urinary cGMP/creatinine ratios ≥ 120 μM/mM before CM application are a significant predictor of MARE (Table 4).

**Table 4:** Multivariate logistic regression analysis for predictors of MARE.

Variables	B	S.E.	Wald	P	Exp(B)	95% C.I. for EXP(B)	
						Lower	Upper
**Age**	0.014	0.030	0.218	0.640	1.014	0.957	1.075
**Diabetes mellitus**	-0.508	0.591	0.740	0.390	0.602	0.189	1.915
**Chronic Kidney Disease**	-1.250	0.961	1.692	0.193	0.286	0.044	1.884
**Anemia**	1.488	0.556	7.169	0.007	4.427	1.490	13.154
**Congestive heart failure**	0.939	0.549	2.923	0.087	2.557	0.872	7.499
**Contrast volume**	-0.014	0.404	0.001	0.973	0.986	0.447	2.175
**Urinary cGMP/creatinine** **ratio ≥ 120** **μM/mM** **before CM application**	1.173	0.559	4.410	0.036	3.233	1.081	9.666

cGMP: urinary cyclic guanosine monophosphate, uCr: urinary creatinine, MARE: major adverse renal event, Exp(B): Odds Ratio, p<0.05 –is statistically significant.

In analogy to cGMP/Cr ration in Table 3, we calculated serum creatinine and GFR in each group divided by death, dialysis or MAREs, but did not see any statistically significant difference between the patients who developed MARE, needed dialysis or died during the follow up and those who did not (Table 5).

**Table 5:** GFR (in ml/min/1.73m^2^) before, 24 hours after and 48h after contrast media application.

		GFR before CM			GFR 24hrs after CM			GFR 48hrs after CM		
		N	M ± SD	p	N	M ± SD	p	N	M ± SD	p
**MARE**	No	271	68.07 ± 30.96	0.14	250	72.19 ± 32.23	0.12	85	71.09 ± 30.03	0.001
	Yes	18	62.12 ± 40.38		16	61.91 ± 50.59		8	14.04 ± 23.20	
**Dialysis**	No	277	68.80 ±31.72	0.06	255	71.35 ± 32.77	0.03	88	72.18 ± 30.69	0.003
	Yes	12	55.85± 31.90		11	17.51 ± 39.14		5	11.27 ± 3.94	
**Death**	No	280	68.43 ± 32.57	0.09	258	71.51 ± 33.47	0.06	87	71.09 ± 32.29	0.002
	Yes	9	63.11 ± 29.9		8	59.69 ± 46.97		6	24.04 ± 23.21	

GFR—glomerular filtration rate according to the MDRD formula, MARE—major adverse renal event; N—number of patients, M—median, SD—standard deviation, p—significance according to MANOVA, p<0.05 is statistically significant.

## Discussion

To our knowledge, this is the first study demonstrating that urinary cGMP/creatinine ratio ≥ 120 μM/mM is predictive for death, the need of emergent dialysis or MARE during 90 days of follow-up in patients with high risk of developing contrast-induced renal failure after CM application for coronary angiography.

We chose 90 days follow-up because this timeframe was previously reported as critical for development of adverse events among patients with AKI [31–33]. Our results are consistent with data on increase of urinary cGMP in patients with diabetes. Comparable with our study, the authors did not find any correlation between creatinine clearance and clearance of cGMP [34].

Hypoxia is an early sign and trigger of progression of diabetic nephropathy [35]. Recent clinical data reported decreased renal oxygenation in patients with CKD. Indeed, decline in renal oxygenation precedes matrix accumulation *in vivo* suggesting that hypoxia may influence both initiation and promotion of fibrosis and that chronic hypoxia is a final common condition of end-stage renal disease [36]http://www.ncbi.nlm.nih.gov/pubmed/?term=Chronic+hypoxia+as+a+mechanism+of+progression+of+chronic+kidney+diseases%3A+from+hypothesis+to+novel+therapeutics. Neylon et al. showed that hypoxia increases urinary cGMP without changes in GFR and absolute sodium excretion [37]. In addition, it was previously reported that the selective activation of soluble guanylate cyclase (sGC) with subsequent rise in cGMP concentration causes potent systemic and renal vasodilating effects, unloads the heart, increases cardiac output, and preserves GFR and sodium and water excretion in experimental chronic heart failure [38].

In contrast to this data, some studies report significantly lower cGMP concentrations in urine in patients with CKD [19]. Additionally, a correlation between decline of urinary cGMP and decrease in diuresis, renal blood flow, glomerular filtration rate and fractional sodium excretion has been reported [39]. In line with our results, it was recently reported that CIN was a significant predictor of subsequent renal events after cardiac catheterization and CIN and anemia were associated with increased risk for worse long-term clinical outcome, especially when both were present [40].

Experimental inhibition and stimulation of renal cGMP was studied in a rodent model of acute and chronic renal failure. Pretreatment of animals with cGMP-specific phosphodiesterase inhibitor accelerated renal recovery after ischemia due to stimulation of regional renal blood flow [41]. Enhancement of renal cGMP levels by administration of sGC stimulator BAY 41–2272 to animals with a progressive renal fibrosis at one week after induction of anti-Thy-1-induced chronic glomerulosclerosis, significantly limited tubulointerstitial fibrosis and preserved renal function [42]. In addition, sildenafil treatment significantly increased urinary cGMP excretion in OLETF diabetic rats and attenuated diabetic nephropathy by decreasing albuminuria, attenuating glomerular hyperfiltration, decreasing glomerular hypertrophy and reducing the glomerulosclerosis score [43, 44]. Furthermore, daily administration of sildenafil initiated immediately after renal ablation resulted in stabilization of creatinine levels, prevention of hypertension, reduction in proteinuria and increase of urinary cGMP excretion in 5/6 nephrectomized rats [45].

Reports on the origin of urinary cGMP are controversial. In clearance studies in rats, urinary cGMP was primarily of renal cellular origin [46]. cGMP clearance in healthy pregnant women is increased in the setting of decreased plasma cGMP levels, indicating increased nephrogenic cGMP production [47]. Studies on 3H-labeled cGMP in dogs showed that a significant amount of urinary cGMP is derived from plasma by tubular secretion [48]. A study on renal clearances of plasma cGMP in humans using tritium-labeled cyclic nucleotides showed that plasma was the source of virtually all of the cGMP excreted with urine [49]. Jakob et al. reported that there was no significant correlation between urinary concentrations of cGMP and creatinine [19].

An increased death rate of patients with higher urinary cGMP/creatinine ratios may be at least partially explained by the fact the patients in our cohort had symptoms of coronary heart disease as an indication for coronary angiography. In patients with heart failure, cGMP concentrations in urine were significantly higher than in healthy volunteers [50]. However, according to New York Heart Association there is no relationship between cGMP concentrations in urine and severity of heart failure functional classes. In plasma, by contrast, there was a significant direct correlation between cGMP concentrations and the severity of heart failure [19]. In addition, ROC analysis showed no significant correlation between plasma cGMP and cumulative (24-month) all-cause mortality in patients with myocardial infarction [51].

In the recent systematic review and meta-analysis, Klein et al. identified the most promising biomarkers of risk for renal replacement therapy among patients with acute kidney injury. Altogether, nine urinary biomarkers were eligible for meta-analysis: NGAL, urinary interleukin 18 (IL18), Kidney injury molecule-1 (KIM-1), urinary N-acetyl-beta-d-glucosaminidase (NAG), Tissue inhibitor of metalloproteinases 2 (TIMP-2), insulin-like growth factor-binding protein-7 (IGFBP7), the fractional excretion of sodium (FeNa) T, urinary cystatin C and urinary output [52]. The authors underlined the moderate quality of the studies, small sample sizes and luck of standardization with risk of bias and confounding suggesting a need for further research in this area.

## Conclusion

Urinary cGMP/creatinine ratios of more than 120 μM/mM could be a promising biomarker for prediction of death, dialysis or MARE during 90 days of follow-up in patients with impaired but remnant kidney function or diabetes after coronary angiography.
